# Associations between inflammatory biomarkers, symptoms, treatment toxicities and quality of life in people with advanced cancer receiving systemic anti-cancer treatments: a systematic review

**DOI:** 10.1007/s00520-026-10922-5

**Published:** 2026-07-01

**Authors:** Helen Andersen, Wei-Hong Liu, Patsy Yates, Morgan J. Farley, Kim E. Alexander

**Affiliations:** 1 Cancer, Blood Disorders and Respiratory Service, Gold Coast Health Service, Queensland Health, Gold Coast, QLD Australia; 2 Graduate Research Centre, Queensland University of Technology, Brisbane, Australia; 3 Centre for Healthcare Transformation, School of Nursing, Queensland University of Technology, Brisbane, Australia; 4 Cancer and Palliative Care Outcomes Centre, School of Nursing, Queensland University of Technology, Brisbane, Australia; 5 Faculty of Health, Human Performance Research Centre, INSIGHT Research Institute, University of Technology Sydney (UTS), Sydney, NSW Australia; 6 Cancer Care Services, Metro North Health, Queensland Health, Brisbane, Australia

**Keywords:** Advanced cancer, Neutrophil-lymphocyte ratio, Platelet-lymphocyte ratio, Monocyte-lymphocyte ratio, Symptoms, Toxicity

## Abstract

**Purpose:**

Examine the quality of evidence relating to the association of routine inflammatory biomarkers neutrophil-lymphocyte ratio (NLR), platelet-lymphocyte ratio (PLR) and monocyte-lymphocyte ratio (MLR) with symptoms, treatment toxicities, and quality of life (QoL) in people with advanced cancer.

**Methods:**

Searches were conducted in MEDLINE, Embase, CINAHL and the Cochrane database to identify studies that investigated associations between NLR, PLR and MLR with symptoms, toxicities and QoL in people with advanced cancer. The evidence was graded as good, fair or poor through the application of the National Institute of Health (NIH) quality assessment tool. A narrative approach was used for data synthesis.

**Results:**

Of the 38 studies reviewed, NLR was the most frequently investigated biomarker. Thirteen studies found no association between NLR and symptoms or treatment toxicities. Of the 25 that did, five provided good quality evidence linking NLR to immune-related adverse events (irAEs), chemotherapy-related haematological toxicities and insufficient oral intake. However, NLR cut-off values varied across studies. Ten studies reported an association between PLR and symptoms or treatment toxicities whilst six studies found no associations; one study found no association with MLR and symptoms or treatment toxicities.

**Conclusion:**

Further research is needed to establish specific cut-off values of NLR to increase the utility of this biomarker in clinical practice. PLR and MLR warrant further investigation for association with symptoms, treatment toxicities and QoL.

## Introduction

Advances in therapeutic interventions have increased overall survival rates for people with advanced cancer; however, this is sometimes accompanied by increased symptom burden and reductions in quality of life (QoL). The use of multiple lines of treatment, longer durations of time spent on treatment and the inclusion of emerging therapies (e.g. immunotherapies) means management of symptoms and side effects is imperative for survivorship care [1, 2].

In the advanced cancer setting, treatment aims to extend survival whilst preserving people’s QoL. The identification of biomarkers associated with symptom experiences could enable a deeper understanding of the underlying mechanisms contributing to symptom burden and provide insights into potential management strategies. These biomarkers could also assist in identifying individuals at higher risk of adverse symptoms and treatment-related side effects, thus supporting clinicians to provide tailored symptom monitoring, proactive intervention and supportive care. Collectively, this information can lead to improved treatment tolerance, reduced symptom burden and improved QoL for people with advanced cancer.

To date, various inflammatory markers such as C-reactive protein (CRP), tumour necrosis factor (TNF) and interleukin (IL)−1, IL–6, and IL–10 have been associated with the presence of symptoms including anorexia, dyspnoea, fatigue, pain and sleep disturbance in people with advanced cancer [3–8]. Previous research has also demonstrated C-reactive protein (CRP) as the primary marker of systemic inflammation associated with symptom burden [3, 6, 7, 9–13]. However, the translation of this evidence into clinical practice is challenging, given that CRP is not routinely performed or recommended in Australia at baseline and during common anti-systemic treatments such as immunotherapy [14–17]. The exception for baseline CRP testing would include people with pre-existing autoimmune diseases [15]. Some guidelines recommend CRP testing if specific irAEs are suspected during immunotherapy including colitis [15], cardiac toxicity [16] and neurological toxicity [16, 17]. There is a consensus that CRP testing should be done if musculoskeletal toxicity is suspected [14–17]. Routine CRP measurement may be impeded by the extra cost associated with testing. Furthermore, CRP concentrations can be highly variable and easily influenced by transient injuries or infections [18]. This variability can affect the clinical significance of the CRP, especially if it is taken as a single measurement.

Other more complex inflammatory markers have also been investigated in relation to symptom burden, including erythrocyte sedimentation rate (ESR), tumour necrosis factor (TNF)-α and transforming growth factor (TGF)-β, IL-1β, IL-2, IL-4, IL–8, IL–12, and IL–18. Testing of these biomarkers presents similar challenges and costs to CRP testing. [6, 8, 12]. The identification of other more accessible biomarkers could therefore have potential to improve treatment outcomes and QoL for people undergoing treatment for advanced cancer.

Full blood counts (FBC) are routinely collected on people receiving systemic treatment for advanced cancer. Inflammatory blood biomarkers that can be calculated from an FBC include neutrophil-lymphocyte ratio (NLR), platelet-lymphocyte ratio (PLR) and monocyte-lymphocyte ratio (MLR). Higher ratios indicate an imbalance between inflammation and immunity resulting in higher inflammatory levels and lower adaptive immune responses promoting tumour growth and metastasis. The NLR is recognised as a prognostic indicator in many solid tumour cancers. NLR > 5 indicates a poorer prognosis [5, 19–26]. An elevated NLR often indicates higher tumour burden or more aggressive disease in some solid tumour cancers. In other chronic conditions including COVID and chronic pulmonary disease, a higher NLR has been associated with exacerbation of disease and increased risk of mortality [27, 28]. Furthermore, NLR has been associated with CRP concentrations in some clinical populations [29, 30].

A growing body of evidence suggests that FBC-derived measures of systemic inflammation may be associated with symptoms and treatment toxicities in the advanced cancer adult population, therefore potentially serving as an accessible and cost-effective option in identifying individuals at higher risk of poorer outcomes, warranting early intervention. Despite this, there are currently no systematic reviews and meta-analyses that synthesise and evaluate the relationships between FBC-derived measures of systemic inflammation and symptoms, treatment toxicities and QoL in adult advanced cancer populations. Two systematic reviews and meta-analyses have examined the association of NLR and other biomarkers with irAEs [31, 32]. Neither of these reviews included symptoms or QoL [31, 32]. This systematic review aims to evaluate the associations between NLR, PLR and MLR with symptoms, treatment toxicities and QoL in adult advanced cancer populations.

## Methods

### Protocol and registration

The protocol for this systematic review was prospectively registered on Prospero CRD42022297756. This review protocol was developed aligning with the Preferred Reporting Items for Systematic Review and Meta-analyses (PRISMA) guidelines [33].

### Search strategy

Electronic searches were conducted in MEDLINE, Embase, CINAHL and the Cochrane database to identify studies published up to October 2024 investigating associations between inflammatory blood biomarkers from a routine blood count with symptoms, toxicities or QoL. Specific inflammatory markers included NLR, PLR and MLR, derived from a FBC. Search terms included keywords, phrases, MESH subject headings or equivalents dependent on the database (e.g. advanced or metastatic cancer/neoplasm/solid tumour, neutrophil to lymphocyte ratio, NLR, platelet to lymphocyte ratio, PLR, monocyte to lymphocyte ratio, MLR, symptoms, symptom assessment, signs and symptoms, drug-related side effects and adverse reactions, toxicity, adverse effects, adverse events and QoL).

### Eligibility criteria

Studies were included if they were an adult population (≥ 18 years) with locally advanced or metastatic solid tumour cancer, on systemic anti-cancer treatment, and reported inflammatory blood markers only from a FBC including NLR, PLR and MLR that were directly correlated with patient-reported or clinician-reported symptoms, treatment toxicities or QoL. Measurements of outcomes or symptoms included timing of onset, frequency, duration, distress or severity. They could be reported as prevalence, incidence, risk, grade or severity. Only studies in English that were fully published and any quantitative design were included.

Studies were excluded if the population was < 18 years old, in populations other than advanced cancer, haematological cancer, or were not receiving systemic anti-cancer treatment for locally advanced or metastatic cancer, inflammatory markers not measured using FBC blood parameters or using different blood tests that are not taken from a full blood count or reported inflammatory markers in other conditions, not cancer. Case reports/series, qualitative studies, unpublished studies and abstracts were excluded.

### Study selection and data extraction, analysis and quality appraisal

Search results were imported to Endnote X7 software, and any duplicates were removed. Results were then exported to a specific online systematic review platform (Covidence). Three reviewers (HA, MF and WL) conducted independent screening of titles and abstracts, followed by a full-text review. If the two reviewers disagreed, a third reviewer (PY) was consulted. Data, including study design, setting, characteristics of study population, reported associations between NLR, PLR, MLR and symptom/treatment toxicity and QoL outcomes, were extracted using a predesigned Excel table and performed independently by two reviewers (HA and WL). Consultation with a third reviewer occurred if there were any unresolved discrepancies (PY).

Two reviewers (HA and WL) performed quality assessment independently using the National Institutes of Health (NIH) tool [34]. Reviewers rated the studies as good, fair or poor quality based on assessments of study design, methodology and risk of bias, including factors such as study population and selection, sample size justification, exposure and outcome assessment, follow-up and confounding adjustment. Disagreements were resolved through a systematic and collaborative process between authors to reconcile differing opinions to reach agreement. Studies were not excluded based on poor quality ratings; instead, results were summarised across all included studies regardless of quality.

Due to the heterogeneity of studies, a narrative synthesis was used to summarise study findings instead of meta-analysis. We grouped studies in which outcome data were reported at the same level, such as by prevalence or incidence rate, by severity or grade level, or by risk of onset. The scope, as well as the differences and similarities among the studies, was systematically described.

## Results

**Fig. 1:**
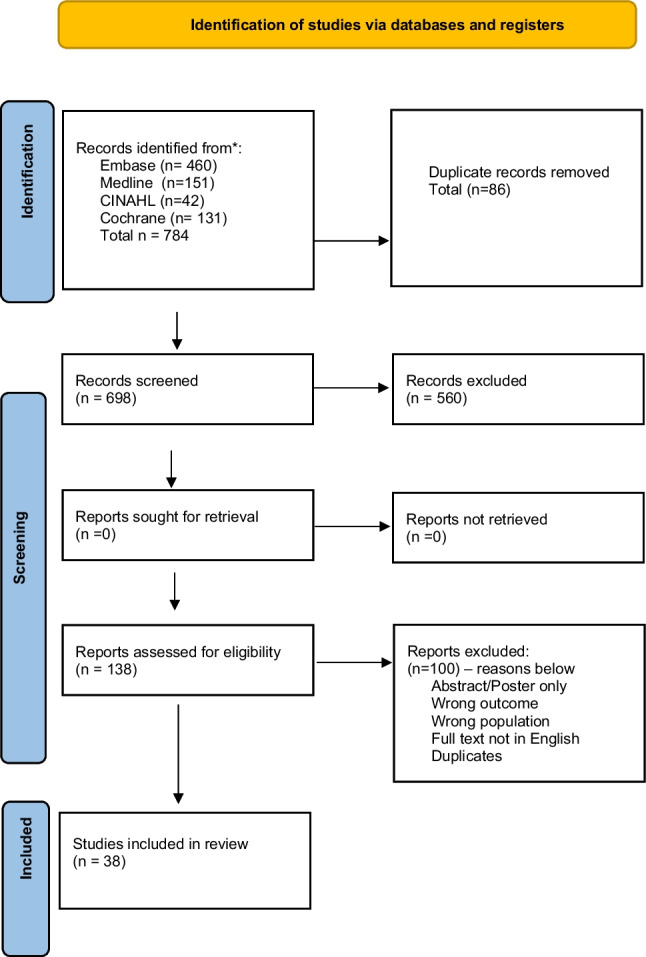
PRISMA flow chart for the search and study selection process

A total of 38 articles were included for review, as identified in Fig. 1.

### Characteristics of included studies

Most studies were retrospective (*n* = 31) [35–65], four were prospective [66–69] and the remaining included a cross-sectional study [70] a nested case–control study [71] and a post hoc analysis of a randomised controlled trial [72]. Twenty-five studies were conducted in Asian countries [38–40, 43–47, 49, 51–56, 58, 61–65, 67–69, 71], five in Europe [41, 42, 48, 57, 59], four in the Americas [37, 60, 66, 70], and one in Canada [50] and three were multinational studies [35, 36, 72]. Eighteen studies included people diagnosed with advanced lung cancer [36, 41, 43, 44, 46–48, 50, 51, 56–59, 61–63, 66, 70], nine upper gastrointestinal and colorectal cancers [38–40, 45, 54, 55, 65, 67, 68], three genitourinary cancers [42, 49, 72], two head and neck cancers [52, 69], and one melanoma [60], and five studies included various cancer types [35, 37, 53, 64, 71].

In relation to biomarkers and symptoms or toxicities, NLR was included in all 38 of the studies, PLR was included in 16 of the studies [38, 40, 42, 44–46, 48, 50–53, 57, 59, 61–66, 71] and MLR was included in one of the studies [45]. PLR was included in 3 other studies; however, it was not investigated in relation to symptoms or toxicities [46, 63, 64]. NLR was measured as a continuous variable in 10 of the studies [37, 40, 47, 51, 54, 60, 62, 65, 67, 70, 73], and a cut-off level for NLR was used in 28 out of the 38 studies [35, 36, 38, 39, 41–46, 48–50, 52, 53, 55–59, 61, 63, 64, 66, 68, 69, 71, 72]. NLR was classified into “high” and “low” categories, with cut-off values varying significantly across the 28 studies, from as low as 2.30 [44] to as high as 8.58 [53]. The most commonly used cut-off for NLR was 5.00 (*n *= 8) [35, 39, 41, 45, 58, 59, 61, 66]. Cut-off values were determined using various methods, including receiver operating characteristic (ROC) analysis, references to previous literature, median values, maximally selected rank statistics based on baseline data and logistical regression analysis. In some cases, the method was not specified. Among the 16 studies that reported PLR, 11 specified a cut-off value [38, 42, 44–46, 48, 50, 52, 53, 57, 59, 63, 64, 66], which varied significantly, from 135 [45] to 441 [50]. The methods used to determine these cut-offs included ROC analysis, references to previous literature, median values, maximally selected rank statistic and in some cases were unspecified. The MLR cut-off used in one study was 0.31. Blood tests were assessed prior to outcome measures in all 38 studies. Twenty four studies included a single blood test timepoint at baseline prior to commencement of treatment [36, 37, 39, 41, 42, 45–53, 55–58, 62–64, 66, 68, 71, 73] and 14 studies included multiple timepoint blood tests [35, 38, 40, 41, 43, 44, 51, 54, 59–61, 65–70, 72].

### Outcome measures

Seventeen studies investigated symptoms or toxicities as primary outcomes, whilst the remaining studies assessed these as secondary outcomes. Twenty-four (62%) of the 38 studies investigated the association of blood biomarkers with irAEs. Seven studies reported the relationship with chemotherapy toxicities and two with tyrosine kinase inhibitors (TKI) toxicities. Two studies investigated depression, whereas the remaining investigated weight loss, sarcopenia and reduced oral intake, respectively. Twenty-three studies used validated clinician assessment tools to measure outcomes, three used specific clinical parameters and ten did not report their outcome measurement methods, though their description suggested clinician-based assessments. Only two of the 38 studies used validated patient-reported outcome measures, which included the Self-rating Depression Scale (SDS) and the Hospital Anxiety Depression Scale (HADS-D). No studies included QoL as an outcome.

### Associations between NLR symptoms and toxicities

Table 1 summarises NLR results. Twenty-five of the 38 studies found an association between NLR and symptoms or toxicities. A higher NLR was associated with increased incidence of irAEs [44, 63, 64, 71], risk of grade III–IV anaemia from chemotherapy [52], severe non-haematological chemotherapy toxicity [39], baseline hypoalbuminaemia in patients prior to chemotherapy [66], sarcopenia [59], insufficient oral intake during chemotherapy [55], lower risk of grade ≥ 3 neutropenia on cabazitaxel chemotherapy [72], weight loss [37] and increased incidence of paronychia and diarrhoea with tyrosine kinase inhibitors (TKIs) [48]. A lower NLR was associated with increased incidence of irAEs in 10 studies [36, 41, 42, 46, 51, 53, 57, 58, 61, 65] and grade ≥ 3 haematologic toxicity with chemotherapy and radiation in one study [38].

**Table 1:** Summary of results NLR

Author year Country	Quality	Sample size (M/F)	Study design	Study aims	Cancer type and treatment type	Blood collection timepoints	Blood parameters/cut off	Outcome	Results
Egami et al. 2021b Japan	Fair	92 (64/28)	Retrospective multicentre	Analyse correlation of NLR, PLR and MLR with overall response rate, disease control rate and PFS Secondary endpoints included above blood correlation with OS and irAE's	Non-small cell lung cancer Pembrolizumab (immunotherapy)	Baseline and 3 weeks after initiation of immunotherapy	NLR 2.3	Early onset irAEs	Baseline NLR > 2.3 significantly associated with an increased risk of onset of any irAEs OR: 5.99, 95% CI 1.73–20.74, *p* = 0.005
Zhang et al. 2022 China	Poor	142 (75/67)	Retrospective single site	Explore associations between immune nutrition related peripheral blood markers including NLR/PLR and others and prognosis	Any cancer PD-L1 negative and MSS Immunotherapy	Baseline	NLR 3.18	irAEs	Baseline NLR higher in people experiencing irAEs (*p* < 0:001)
Zhao et al. 2022 China	Good	168 (132/36)	Nested case control study	Investigate the demographic, clinical and laboratory markers that are associated with higher ir-SAE risks	Any cancer Immunotherapy	Baseline	NLR groups G1 (< 3) G2 (3–6) G3 (> 6)	Grade ≥ 3 irAEs	Baseline NLR independent predictive factor for grade ≥ 3 irAEs (per SD increment OR: 1.16, 95% CI 1.0–1.32, *p* = 0.019) Participants with a higher NLR in group 3 (NLR > 6) versus group 1 (NLR < 3) had a fourfold increased risk of grade ≥ 3 irAEs
Liu and Lin 2019 Taiwan	Poor	40 (38/2)	Retrospective single site	Identify possible demographic and laboratory factors that might influence the chance of more severe acute toxicities and better response	Head and neck cancer Docetaxel combined chemotherapy	Baseline	NLR 3.5	Severe acute treatment toxicities	Baseline NLR ≥ 3.5 significantly associated with the risk of grade III–IV anaemia (*p* = 0.006)
Chan et al. 2024 Hong Kong	Fair	158 108/50	Retrospective single site	Examine associations between skeletal muscle mass and chemotherapy toxicities and survival and define a SMI cutoff point associated with the risk of severe toxicities and survival Risk factors of severe chemotherapy toxicities also performed	Gastric cancer Chemotherapy	Baseline	NLR 5	Severe treatment toxicities	Baseline NLR ≥ 5 independent predictor of severe non haematological toxicities OR 3.13 95% CI 1.40–6.98 *p* = 0.005
Arrieta et al. 2010 Mexico	Poor	100 (53/47)	Prospective cohort study	Investigate associations of malnutrition and albumin serum levels with the occurrence of chemotherapy-induced toxicity	Non-small cell lung cancer Paclitaxel-cisplatin chemotherapy	Baseline	NLR 5	Hypoalbuminaemia and chemotherapy toxicities	Baseline NLR ≥ 5 was associated with baseline hypoalbuminemia (mean ranks, 55.7 vs. 39 *p* = 0.006)
Petrova et al. 2020 Bulgaria	Poor	167 (106/61)	Retrospective cohort study multicentre	Investigate indicators associated with development of hyper progressive disease	Non-small cell lung cancer Pembrolizumab immunotherapy after first line chemotherapy	Prior to chemotherapy and again prior to immunotherapy	NLR 5	Sarcopenia	Pre immunotherapy NLR higher in people with sarcopenia compared to those without sarcopenia (7·9 ± 3·2 versus 3·6 ± 2·3) *p* < 0.0001
Ogata et al. 2021 Japan	Good	589 (M/F not reported)	Retrospective cohort, single site	Clinicopathological systemic inflammatory response and nutritional biomarkers for predicting the efficacy of nivolumab	Gastric cancer Chemotherapy	Baseline	NLR 3	Insufficient oral intake	Baseline NLR > 3 associated with insufficient oral intake in 2nd line and 3rd line chemotherapy OR 3.09 CI 1.65–5.76 *p* < 0.001 and OR 2.65 CI 1.32–5.31 *p* 0.006
Meisel et al. 2016 Multiple countries	Good	755 (all male)	Post hoc analysis of phase III randomised trial	Primary: explore the prognostic role of grade ≥ 3 neutropenia during cabazitaxel therapy on overall survival Secondary: evaluate the effect of grade ≥ 3 neutropenia on progression-free survival (PFS)	Prostate cancer Cabazitaxel chemotherapy	Pre D1, D8, D15 of each cycle of chemotherapy	NLR 3	Grade ≥ 3 neutropenia	Baseline high NLR ≥ 3 associated with significantly lower occurrence of grade ≥ 3 neutropenia during cabazitaxel therapy (88.8% versus 75.3%, *p* = 0.002)
Barker et al. 2020 USA	Poor	50 (32/18)	Retrospective cohort Single site	Determine if elevation in NLR associates with greater weight loss, cachexia and lower vitamin D concentrations in advanced cancer patients	Colon, lung, and prostate Specific treatment not stated	Baseline	NLR 3.15	Body weight loss and cachexia	Higher baseline NLR associated with weight loss
Jokic et al. 2021 Serbia	Poor	101 (35/66)	Retrospective	Assess the prognostic and predictive value of various blood test parameters	Non-small cell lung cancer Tyrosine kinase inhibitors	Baseline	NLR 2.9	Treatment toxicity	Baseline NLR > 2.9 more often presented with toxicity, especially paronychia and diarrhoea *p* = 0.060 and *p* = 0.035
Anpalakhan et al. 2023 Multiple countries	Fair	308 (171/137)	Retrospective observational cohort study sub analysis multicentre	Describe the frequency, type and severity of irAEs observed in patients and association with survival outcomes and progression-free survival PFS Secondary endpoints included assessing for possible clinical factors influencing the likelihood of irAEs	Non-small cell lung cancer Immunotherapy	Baseline	NLR 4	irAEs	Baseline NLR < 4 potential predictor of development of grade 1–2 irAEs compared to any grade irAEs (*p* = 0.013 and *p* = 0.018)
Daniello et al. 2021 Germany	Poor	939 (561/368)	Retrospective cohort, single site	Investigate therapeutic and prognostic implications of immune-related adverse events in advanced non-small-cell lung cancer	Non-small cell lung cancer Immunotherapy with or without chemo	Baseline	NLR 5	irAEs	Lower baseline NLR < 5 significantly associated with occurrence of irAEs (*p* < 0.001)
Dionese et al. 2024b Italy	Fair	119 (83/36)	Retrospective single site	Investigate potential association between three baseline systemic inflammation indexes and the development of irAEs in a real-world cohort of patients	Urothelial cancer Immunotherapy	Baseline	NLR 3.52	irAEs	Baseline NLR < 3.52 significantly associated with a higher risk of developing irAEs (OR = 3.89, 95% CI = 1.14–14.38, *p* = 0.03)
Fujimoto et al. 2023 Japan	Fair	315 (240/75)	Retrospective multicentre	Investigate the risk factors for the occurrence of irAEs	Non-small cell lung cancer Immunotherapy and chemotherapy	Baseline	NLR 2.79	irAEs	Baseline NLR < 3 associated with occurrence of irAEs but did not correlate with time of onset or severity OR 2.91, 95% CI 1.35–6.27, *p* < 0.01
Liu et al. 2021 China	Poor	150 (118/32)	Retrospective cohort, single site	Explore influences of inflammation related peripheral blood markers NLR and PLR on anti PD-1 inhibitor induced irAEs	Non-small cell lung cancer Immunotherapy	Baseline	No cut off	irAEs	Baseline lower levels of NLR associated with grade 3–4 irAE’s *p* = 0.023 NLR significantly decreased during the treatment cycle when irAEs occurred *p* = 0.017
Ma et al. 2022 China	Fair	95 (66/29)	Retrospective	Analyse associations between irAEs and anti-PD-1/PD-L1 inhibitor responses and evaluate the predictive values of serum biomarkers with respect to the risk of irAEs	Any cancer Immunotherapy	Baseline	NLR 8.58	irAEs	Baseline low-NLR important predictor of irAEs OR: 0.196, 95% CI 0.038–1.000 *p* = 0.0499 Univariate analysis only
Peng et al. 2020 China	Fair	102 (87/15)	Retrospective single site	Explore associations between inflammation-related peripheral blood markers (NLR, LDH, and PNI), and outcome and onset of irAEs	Non-small cell lung cancer Immunotherapy	Baseline	NLR 5	irAEs	Baseline low NLR independent predictor for the onset of irAEs (OR = 0.04, 95% CI 0.01–0.13) (*p* < 0.001)
Shi et al. 2021 China	Fair	103 (68/35)	Retrospective cohort, single site	Investigate the relationship between pretreatment peripheral blood cell parameters and circulating cytokines and the objective response rate (ORR), onset of irAEs, progression-free survival (PFS) and overall survival (OS)	Non-small cell lung cancer Immunotherapy with or without other systemic therapies	Baseline and 6–8 weeks post immunotherapy initiation	NLR 5	irAEs	Baseline NLR lower in people with irAEs compared to those without irAEs (2.915 vs. 3.990, *p* = 0.007)
Zhang et al. 2022 China	Good	243 (172/71)	Retrospective cohort, single site	Investigate the value of baseline peripheral blood biomarkers for predicting treatment outcomes and irAEs	Oesophageal, gastric or colon cancer Immunotherapy	Baseline and 2–3 weeks post immunotherapy initiation	No cut-off	irAEs	Lower cycle 2 NLR associated with increased incidence rate of any grade irAE (OR = 0.875, 95% CI 0.787–0.972)
Cai et al. 2020 China	Good	311 (247/64)	Retrospective	Evaluate association between haematological markers and mortality and adverse events	Oesophageal cancer Neoadjuvant chemoradiation	Baseline and 21 days post neoadjuvant chemoradiation	NLR 2.77	Haematological and non-haematological toxicities	Baseline lower NLR hazard ratio predictor of grade ≥ 3 haematologic toxicity (0.39; 95% CI 0.19–0.77; *p* = 0.007)
Pavan et al. 2019 Italy	Poor	184 (125/59)	Retrospective cohort, single site	Evaluate association between peripheral blood markers and onset of irAE’s Secondary aims evaluate NLR and PLR on outcome in terms of radiological response, overall survival and PFS	Non-small cell lung cancer Immunotherapy	Baseline	NLR 3	irAEs	Baseline low NLR associated with irAEs in 2.2 (95% CI 1.1–4.1; *p* = 0.018) univariate analysis only
Wang et al. 2024 China	Poor	206 (164/42)	Retrospective single site	Prediction of the efficacy of first-line anti- PD-1 therapy and the assessment of irAE risk in advanced NSCLC patients	Non-small cell lung cancer Chemotherapy and immunotherapy or chemotherapy alone	Baseline	NLR 2.8	irAEs	Baseline NLR correlates with occurrence of irAEs (chi square tests) 11.252 0.001
Li et al. 2014 China	Poor	56 (30/26)	Prospective	Explore the exact relationship between advanced age and depression Explore immune parameters to identify a subjective biomarker	Colorectal cancer Chemotherapy	First 2 days of study and again dependent on SDS score	Not defined	Depression	NLR was positively correlated with Self Rating Depression Score (SDS) (*r* = 0.634, *p* < 0.05)
McFarland et al. 2020 USA	Poor	96 (37/58)	Cross sectional	Association of tumour mutation burden with depression and relationship moderation by immune system activation	Lung cancer Any active systemic treatment	Multiple timepoints throughout treatment	No cut off	Depression	NLR taken at any timepoint throughout treatment was associated with depression (*r* 5 0.21; *p* 5.017) Univariate analysis only
Ameratunga 2018 UK, Spain, Australia	Fair	165 (91/74)	Retrospective cohort, multicentre	Primary aim to evaluate relationship of baseline NLR with response, survival and toxicity Secondary aim to evaluate longitudinal kinetics of the NLR	Any solid tumour cancer Immunotherapy	Prior to each cycle of immunotherapy	NLR 5	irAEs	Baseline NLR not associated with any grade immune toxicity and grade 3–4 toxicity (OR = 0.98, 95% CI 0.91–1.05, *p* = 0.58) (OR = 1.06, 95% CI 0.98–1.15, *p* = 0.13)
Egami 2021a Japan	Fair	171 (113/58)	Retrospective observational multicentre	Identify peripheral blood count data that may be predictive of the development of nivolumab induced irAE’s in a real-world setting	Non-small cell lung cancer Immunotherapy	Baseline and 3 weeks after immunotherapy initiation	NLR 4.3	irAEs	No significant association with an increased risk of early onset irAE’s and NLR > 4.3 adjusted (OR: 0.57, 95% CI 0.30–1.08; *p* = 0.083) No significant association of NLR > 4.3 at 2 weeks after treatment initiation with skin reactions and diarrhoea adjusted OR: 0.79, 95% CI 0.41–1.54; *p* = 0.492
Fan et al. 2021 China	Poor	111 (56/55)	Retrospective cohort (before-after) study	Analyse correlation of NLR, PLR and MLR with overall response rate, disease control rate and PFS Secondary endpoints included above blood correlation with OS and irAEs	Gastric and colorectal cancer Immunotherapy with chemo, radiation or targeted therapy	Baseline	NLR 5	irAEs	No associations between baseline NLR and the risk of irAEs (*p* = 0.107)
Kobayashi et al. 2020 Japan	Poor	44 (34/9)	Retrospective	IrAEs and pembrolizumab efficacy and investigation of factors associated with irAEs	Urothelial cancer Immunotherapy	Baseline	NLR 4	irAEs	Baseline NLR not associated with occurrence of irAEs OR = 1.39, 95% CI 0.30–6.35, *p* = 0.485
Ksienski et al. 2021 Canada	Good	220 (99/121)	Retrospective cohort, single site	Peripheral blood inflammatory markers with overall survival (OS) Clinical risk factors for development of immune related adverse events (irAE) were also explored	Non-small cell lung cancer Immunotherapy	Baseline	NLR 6.4	irAEs	Baseline NLR not significantly associated with irAE's within 6 months or 8 months of pembrolizumab initiation
Namikawa et al. 2020 Japan	Poor	29 (19/10)	Retrospective, cohort single site	Investigate the usefulness of clinicopathological systemic inflammatory response and nutritional biomarkers for predicting the efficacy of nivolumab	Gastric cancer Immunotherapy	Baseline and throughout immunotherapy treatment	No cut off	irAEs	No significant differences in the NLR at any time between the irAE-positive and irAE-negative groups
Sonehara et al. 2022 Japan	Poor	113 (91/22)	Retrospective single site	Examine the predictive factors correlated with the development of irAEs	Non-small cell lung cancer Immunotherapy	Baseline	NLR ranges between irAE and non-irAE groups	irAEs	Baseline NLR not significantly different between patients with irAEs and without irAEs (*p* = 0.240)
Wang et al. 2020 China	Poor	48 (25/23)	Single arm prospective study multicentre	Evaluation of efficacy and safety of apatinib monotherapy	Colorectal cancer Tyrosine kinase inhibitor	Baseline	NLR 4.1	Treatment toxicities/AEs	Baseline NLR not associated with treatment toxicities *p* = 0.075
Xu et al. 2018 China	Poor	546 (418/127)	Prospective single site	Investigate the associations between the occurrence and grade of neutropenia during 1 st cycle immunotherapy and survival Secondary aims were to assess the occurrence of myelosuppression events and post-NLR after 1 st cycle of immunotherapy and explore whether the timing of neutropenia, number of myelosuppression events and high post-NLR could influence survival	Nasopharyngeal Chemo and chemo/rad	Baseline and weekly day 1 and day 21 of chemotherapy	NLR 2.35	Myelosuppression	No significant differences in terms of baseline NRL level between 3 groups: absent, grade 1–2 and grade 3–4 neutropenia
Hou et al. 2024 China	Poor	112 IrAE 14/13 Non IrAE 51/34	Retrospective single site	Explore the predictive value of peripheral blood parameters, including WBC, NLR, sATPCD4 and nATPCD4, for irAEs in advanced NSCLC	Non-small cell lung cancer Immunotherapy	Baseline	No cut off	irAEs	No significant differences were observed in the baseline NLR between the irAEs and non-irAEs groups (*p* = 0.639)
Ozawa et al. 2024 Japan	Fair	199 162/37	Retrospective multicentre study	Evaluate predictive factors for irAE development and prognostic factors associated with chemoimmunotherapy	Non-small cell lung cancer Immunotherapy and chemotherapy	Baseline	NLR 3	irAEs	Baseline NLR ≥ 3 not associated with development of irAEs OR 1.09 CI 0.61–1.96 *p* = 0.764
Pozorski et al. 2023 USA	Fair	183 113/70	Retrospective single site	Elucidate the utility of pretreatment NER as a biomarker of ORR, PFS, OS and risk of high-grade (grade ≥ 3) irAEs Evaluate pretreatment NLR to further validate its ongoing use as a biomarker	Melanoma Immunotherapy	Baseline and 1 month post immunotherapy	No cut off	irAEs	Neither NLR at baseline or 1-month follow up were associated with high-grade irAEs
Chen et al. 2023 China	Fair	190 137/53	Retrospective multisite but only one site for nomogram predicting G4 Lymphopenia	Investigate the relationship between radiation parameters and the risk of G4 lymphopenia Assess whether G4 lymphocyte reduction during CCRT was associated with prognosis. Establish a nomogram for predicting G4 lymphocyte nadir	Oesophageal Chemoradiation	Baseline and weekly throughout chemoradiation	Not defined	Grade 4 Lymphopenia	NLR not included in nonogram to predict G4 lymphopenia

*M* male, *F* female, *NLR* neutrophil-lymphocyte ratio, *PLR* platelet-lymphocyte ratio, *MLR* monocyte-lymphocyte ratio, *SDS* self-rating depression score, *MSS* microsatellite stable, *PD–L1* programmed death-ligand 1, *irAEs* immune-related adverse events, *ir-SAE* immune-related serious adverse event, *OR* odds ratio, *CI* confidence interval, *SD* standard deviation

Only two studies used patient-reported symptoms. One study found that NLR was significantly higher in patients with advanced colorectal cancer who were depressed compared with non-depressed using the self-rating depression scale (SDS) [67]. There was no cut-off for NLR in this study. The second study found a positive association between NLR and depression in patients with advanced lung cancer [70].

Thirteen studies found no association between NLR and symptoms or toxicities [35, 40, 43, 45, 47, 49, 50, 54, 56, 60, 62, 68, 69].

### Associations between PLR, symptoms and toxicities (Table 2)

**Table 2:** Summary of results PLR

Author year Country	Quality	Sample size (M/F)	Study design	Study aims	Cancer type and treatment type	Blood collection timepoints	Blood parameters/cut off	Outcome	Results
Egami et al. 2021b Japan	Fair	92 (64/28)	Retrospective observational multicentre	Analyse correlation of NLR, PLR and MLR with overall response rate, disease control rate and PFS Secondary endpoints included above blood correlation with OS and irAE’s	Non-small cell lung cancer Pembrolizumab (immunotherapy)	Baseline and 3 weeks after initiation of immunotherapy	PLR 165	Early onset irAEs	Pre-treatment PLR > 165 significantly associated with an increased risk of onset of any irAEs adjusted OR: 2.87, 95% CI 1.16–7.08, *p* = 0.022)
Jokic et al. 2021 Serbia	Poor	101 (35/66)	Retrospective	Assess the prognostic and predictive value of various blood test parameters	Non-small cell lung cancer Tyrosine kinase inhibitors	Baseline	PLR 190	Treatment Toxicity	Baseline PLR > 190 more often had paronychia diarrhoea and hyperbilirubinemia *p* = 0.058, *p* = 0.044 and *p* = 0.077
Petrova et al. 2020 Bulgaria	Poor	167 (106/61)	Retrospective cohort study multicentre	Investigate indicators associated with development of hyper progressive disease	Non-small cell lung cancer Immunotherapy after first line chemotherapy	Prior to chemotherapy and again prior to immunotherapy	PLR 174	Sarcopenia	Patients with sarcopenia had significantly higher pre immunotherapy PLR values than patients without sarcopenia 315·9 ± 157·9 versus 168·7 ± 93·8 *p* < 0.0001
Arrieta et al. 2010 Mexico	Poor	100 (53/47)	Prospective cohort study	Investigate associations of malnutrition and albumin serum levels with the occurrence of chemotherapy-induced toxicity	Non-small cell lung cancer Paclitaxel-cisplatin chemotherapy	Baseline	PLR 150	Hypoalbuminaemia and chemotherapy toxicities	Baseline PLR ≥ 150 was significantly related to hypoalbuminemia, development of toxicity grade III/IV and anaemia 58.9 vs. 41.3; *p* = 0.02, 59.3 vs 47 *p* = 0.008 37.9 vs 53.8 *p* = 0.004
Liu and Lin 2019 Taiwan	Poor	40 (38/2)	Retrospective cohort, single site	Identify possible demographic and laboratory factors that might influence the chance of more severe acute toxicities and better response	Head and neck cancer Docetaxel combined chemotherapy	Baseline	PLR 15	Severe acute treatment toxicities	PLR ≥ 15 associated with risk of severe acute toxicity in univariate analysis only
Fan et al. 2021 China	Poor	111 (56/55)	Retrospective cohort (before-after) study	Analyse correlation of NLR, PLR and MLR with overall response rate, disease control rate and PFS Secondary endpoints included above blood correlation with OS and irAEs	Gastric and colorectal Immunotherapy with chemo, radiation or targeted therapy	Baseline	PLR 135	irAEs	Baseline PLR < 135 was associated with a higher probability of irAEs (*p* = 0.028)
Liu et al. 2021 China	Poor	150 (118/32)	Retrospective cohort, single site	Explore influences of inflammation-related peripheral blood markers NLR and PLR on anti PD-1 inhibitor induced irAEs	Non-small cell lung cancer Immunotherapy	Baseline	No cut off	irAEs	Baseline lower levels of PLR associated with grade 3–4 irAE’s group *p* = 0.0016 PLR significantly decreased during the treatment cycle when irAEs occurred *p* = 0.013
Pavan et al. 2019 Italy	Poor	184 (125/59) 79 in control group	Retrospective cohort, single site	Evaluate association between peripheral blood markers and onset of irAE’s Secondary aims evaluate NLR and PLR on outcome in terms of radiological response, overall survival and PFS	Non-small cell lung cancer Immunotherapy	Baseline	PLR 180	irAEs	Multivariate model confirmed low baseline PLR as independent predictive factor for irAEs OR 2.3; 95% CI 1.1–4.8; *p* = 0.027
Shi et al. 2021 China	Fair	103 (68/35)	Retrospective cohort, single site	Investigate the relationship between pretreatment peripheral blood cell parameters and circulating cytokines and the objective response rate (ORR), onset of irAEs, progression-free survival (PFS) and overall survival (OS)	Non-small cell lung cancer Immunotherapy with or without other systemic therapies	Baseline and 6–8 weeks post immunotherapy initiation	No cut-off	irAEs	Patients with irAE’s had lower baseline PLR compared to those without irAEs (159.560 vs. 207.780, *p* = 0.032) Univariate analysis only
Chen et al. 2023 China	Fair	190 137/53	Retrospective multisite but only one site for nomogram predicting G4 Lymphopenia	Investigate the relationship between radiation parameters and the risk of G4 lymphopenia Assess whether G4 lymphocyte reduction during CCRT was associated with prognosis. Establish a nomogram for predicting G4 lymphocyte nadir	Oesophageal cancer Chemoradiation	Baseline and weekly throughout chemoradiation	Not defined	Grade 4 lymphophenia	PLR included in nongram to predict G4 lymphopenia OR 1.007 CI (1.002–1.014) *p* 0.008
Zhao et al. 2022 China	Good	168 (132)	Nested case control study	Investigate the demographic, clinical and laboratory markers that are associated with higher ir-SAE risks	Any cancer Immunotherapy	Baseline	No cut off	Grade ≥ 3 irAEs	Baseline PLR not associated with a higher risk of grade ≥ 3 irAEs
Cai et al. 2020 China	Good	311 (247/64)	Retrospective	Evaluate association between haematological markers and mortality and adverse events	Oesophageal cancer Neoadjuvant chemoradiation	Baseline and 21 days post neoadjuvant chemoradiation	PLR 142.17	Haematological and non-haematological toxicities	PLR not associated with haematological and non-haematological toxicities
Ksienski et al. 2021 Canada	Good	220 (99/121)	Retrospective cohort, single site	Peripheral blood inflammatory markers with overall survival (OS) Clinical risk factors for development of immune related adverse events (irAE) were also explored	Non-small cell lung cancer Immunotherapy	Baseline	PLR 441.8	irAEs	Baseline PLR not significantly associated with irAE’s within 6 months or 8 months of pembrolizumab initiation
Ma et al. 2022 China	Fair	95 (66/29)	Retrospective	Analyse associations between irAEs and anti-PD-1/PD-L1 inhibitor responses and evaluate the predictive values of serum biomarkers with respect to the risk of irAEs	Any cancer Immunotherapy	Baseline	PLR 180.68	irAEs	Baseline PLR not associated with irAEs OR 0.537 CI 0.200–1.440 *p* = 0.216
Dionese et al. 2024b Italy	Fair	119 83/36	Retrospective single site	Investigate potential association between three baseline systemic inflammation indexes and the development of irAEs in a real-world cohort of patients	Urothelial Immunotherapy	Baseline	PLR 194	irAEs	Baseline PLR not associated with risk of developing irAEs
Sonehara et al. 2022 Japan	Poor	113 (91/22)	Retrospective single site	Examine the predictive factors correlated with the development of irAEs	Non-small cell lung cancer Immunotherapy	Baseline	PLR ranges between irAE and non-irAE groups	irAEs	Baseline PLR not significantly different between patients with irAEs and without irAEs (*p* = 0.885)

*M* male, *F* female, *NLR* neutrophil-lymphocyte ratio, *PLR* platelet-lymphocyte ratio, *MLR* monocyte-lymphocyte ratio, *SDS* self-rating depression score, *MSS* microsatellite stable, *PD–L1* programmed death-ligand 1, *irAEs* immune-related adverse events, *ir-SAE* immune-related serious adverse event, *OR* odds ratio, *CI* confidence interval, *SD* standard deviation

Table 2 summarises PLR results. Ten studies found an association between PLR, symptoms and toxicities. A higher PLR was associated with increased incidence of irAEs [44], increased occurrence of paronychia, diarrhoea and hyperbilirubinemia in patients having TKIs [48], sarcopenia [59], anaemia and the development of toxicity grade III/IV in patients treated with chemotherapy [66], grade 4 lymphopenia with chemoradiotherapy (40) and severe acute toxicity with chemotherapy [52]. A lower PLR was associated with increased incidence of irAEs [45, 51, 57, 61]. Six studies found no association between PLR and symptoms or toxicities [38, 42, 50, 53, 62, 71].

### Association between MLR, symptoms and toxicities (Table 3)

**Table 3:** Summary of results MLR

Author year Country	Quality	Sample size (M/F)	Study design	Study aims	Cancer type AND treatment type	Blood collection timepoints	Blood parameters/cut off	Outcome	Results
Fan et al. 2021 China	Poor	111 (56/55)	Retrospective cohort (before-after) study	Analyse correlation of NLR, PLR and MLR with overall response rate, disease control rate and PFS Secondary endpoints included above blood correlation with OS and irAEs	Gastric and colorectal Immunotherapy with chemo, radiation or targeted therapy	Baseline	MLR 0.31	Immune-related adverse events (irAEs)	No associations between MLR and the risk of irAEs (*p* = 0.810)

*M* male, *F* female, *NLR* neutrophil-lymphocyte ratio, *PLR* platelet-lymphocyte ratio, *MLR* monocyte-lymphocyte ratio, *SDS* self-rating depression score, *MSS* microsatellite stable, *PD–L1* programmed death-ligand 1, *irAEs* immune-related adverse events, *ir-SAE* immune-related serious adverse event, *OR* odds ratio, *CI* confidence interval, *SD* standard deviation

Table 3 summarises MLR results. Only one study included MLR. No association between baseline MLR and the probability of irAEs was found. The cut-off point used for MLR was 0.31 [45].

#### Quality appraisal

Overall, six studies were rated good quality [38, 50, 55, 64, 71, 72], thirteen as fair [35, 36, 39, 40, 42–44, 46, 53, 56, 58, 60, 61] and nineteen as poor [37, 41, 45, 48, 49, 51, 52, 54, 57, 59, 62, 64, 66–70, 73–75]. Studies were rated poor quality due to lack of measurement or adjustment for potential confounders, exclusion of missing data, lack of validated outcome measures, small sample sizes, unclear sampling methods and the retrospective nature of some studies.

Studies rated good quality included NLR associations with irAEs, haematological toxicities with chemoradiation and insufficient oral intake during chemotherapy [38, 55, 65, 71, 72]. One good-quality study found no association between NLR and irAEs and a weak association between PLR and irAEs [50]. All the good-quality studies used validated and reliable outcome measures, including recognised specific clinical parameters and CTCAE grading and included potential confounders.

Studies rated fair quality included the association of NLR with increased incidence of irAEs and non-severe haematological toxicities [36, 42, 44, 46, 53, 58]. PLR was associated with irAEs [44, 57] and grade 4 lymphophenia as part of a larger nomogram [40]. Four fair quality studies showed no associations between NLR and irAEs [35, 43, 56, 60].

Twelve studies rated poorly showed associations between blood biomarkers and hypoalbuminemia, development of grade III/IV toxicity and anaemia on chemotherapy, weight loss, sarcopenia, increased risk of paronychia and diarrhoea with TKIs, severe haematological toxicities with chemotherapy and depression [37, 41, 45, 48, 51, 52, 59, 62, 64, 66, 67, 70, 73].

Six of the poorly rated studies found no associations between blood biomarkers and risk of irAEs, TKI treatment toxicities and neutropenia [47, 49, 54, 62, 68, 69].

## Discussion

This systematic review examined the associations between NLR, PLR and MLR with symptoms, toxicities and QoL in people receiving systemic anti-cancer treatments for advanced cancer. To the authors’ knowledge, this was the first of its kind. This review highlighted that NLR is the most common blood biomarker reported in the literature. This is likely due to its previous establishment as a validated prognostic biomarker in this population [5, 19–26]. The established relationship between NLR and disease progression suggests that NLR provides some explanation for the associations identified in this review. Immune-related adverse events were the most common outcome measured, reflecting an increasing interest in exploring associations of more routine blood biomarkers as potential predictors of irAEs.

Whilst the findings from this systematic review identified associations between blood biomarkers, and some symptoms and toxicities, available evidence is currently mixed and paradoxical. Of the studies rated good quality, five demonstrated a significant association between NLR and outcomes, including irAEs, ≥ 3 neutropenia, haematological toxicities and insufficient oral intake [38, 55, 65, 71, 72]. However, two studies observed positive associations between a high NLR and increased risk of serious irAEs and increased risk of insufficient oral intake in 2nd and 3rd line chemotherapy, whereas the remaining three studies observed negative associations. A low NLR was associated with increased risk of haematological toxicities in people having chemoradiation and increased incidence rate of any irAEs. A high NLR was associated with a lower risk of grade ≥ 3 neutropenia during chemotherapy, possibly explained by the association between a high NLR and higher levels of inflammation and lower immune response. The association of high inflammation levels and symptoms in the advanced cancer population is well documented [4–7, 11], providing evidence which is consistent with the findings in this review which identified that higher NLR is associated with increased risk of symptoms and toxicities.

However, a low NLR would normally indicate a more balanced inflammatory immune status in the body. Nine studies identified an association between low NLR and increased incidence of irAEs. This finding is consistent with two other systematic reviews and meta-analyses conducted by Zhang et al. (2023) and Zhou et al. (2023) which reported a significant association between low baseline NLR and increased incidence of irAEs [31, 32]. Zhang et al. (2023) acknowledge that the results of these findings remain unclear [31]. A potential explanation provided by Zhou et al. (2023) is that low neutrophil counts and high lymphocyte counts resulting in a low NLR signify preservation of antitumour effects increasing irAE incidence [32]. It is well documented that the onset of irAEs is associated with better response to immunotherapy treatment which supports the theory that a lower NLR may increase the risk of developing irAEs [76].

Given the variance in outcome measures, cut-off points, treatment and cancer types, drawing comparisons between studies is challenging. The contradictory data observed in this systematic review may be explained, at least in part, due to the majority of studies failing to account for normal ranges. While NLR typically has a normal/healthy range (between 1 and 3) [77] the cut-off points determined by the included studies did not always account for this nuance. Consequently, groups categorised as “high” or “low” may have included individuals with normal NLR values, thus diluting the observed responses and contributing to the variance in results. Notably, Zhao and colleagues accounted for this by dividing the cut-off points into three categories: low (< 3), moderate (3–6) and high (> 6). Their findings revealed that the high NLR group had a fourfold increased risk of immune-related adverse events (irAEs) compared to the low group. This observation supports the theory that a more refined categorisation of NLR could allow for better risk discrimination and potentially explain, at least partially, the discrepancies observed across studies. Future research with consistent cut-off points is required to provide more definitive conclusions.

There were no good quality studies that found an association between PLR, symptoms and toxicities. PLR has not been studied to the same extent as NLR in relation to symptoms and toxicities in people with cancer. This may be, at least in part, due to NLR being an established inflammatory marker in people with solid tumour cancers thus has been prioritised in research over PLR.

Patient-reported outcomes were significantly underrepresented in the studies in this review. Depression was the only patient-reported outcome measure reported within the studies [67, 70]. In these studies, both reported high NLR was associated with depressive symptoms [67, 70]. Early identification and management of symptoms can improve overall QoL, increase survival and improve treatment outcomes [1, 2, 78]. Given a key priority when treating people with advanced cancer is to improve clinical outcomes whilst minimising the impact on QoL, using biomarkers like NLR in clinical practice could have potential to identify people at risk of high symptom burden. This would provide an opportunity to initiate early supportive care interventions which have the potential to improve QoL and clinical outcomes. However, more research is required to examine this hypothesis.

Moreover, such biomarkers would not replace patient-reported outcome measures, nor would they be used as a single measurement of risk. People may not experience any symptoms prior to commencing treatment and baseline biomarkers may contribute to identifying people at risk of higher symptom burden throughout treatment pre-emptively. Patient-reported outcome measures and clinician assessments would remain important to assess ongoing symptoms. Patient-reported measures used at baseline may not identify those likely to develop significant symptom burden or adverse events, which biomarkers may provide.

Blood biomarkers may also be combined with other potential risk factors to contribute to a nomogram tool which involves a calculation or prediction of risk based on certain established risk factors. An example being the Cancer and Aging Research Group Chemotherapy Toxicity Tool (CARG-TT) [79] which provides a percentage risk for grade 3 or greater toxicity from chemotherapy for geriatric oncology patients. This tool also includes certain blood parameters as well as other factors to determine risk assessment.

### Limitations

There are several limitations worthy of consideration. Firstly, this research was focused on markers of systemic inflammation that could be obtained from routine full blood counts, a cost-effective and feasible measurement that is often used in standard oncology care. As such, we acknowledge that there may be other markers of inflammation that may be better prognostic markers of symptoms, treatment toxicities and unfavourable changes in QoL and are worthy of investigation in future research. However, these may be cost-prohibitive and thus limit implementation into standard of care and as such were not within the scope of this review. Secondly, cut-off points for low/high NLR, PLR and MLR varied significantly between studies and often did not include a homeostatic or normal range. This both limited the ability to compare results between studies and may dilute the associations detected. Future research should aim to use consistent evidence-based cut-off values and account for the healthy range within their analyses.

Whilst NLR has clinical utility and is a validated prognostic tool in cancer populations, NLR can be influenced by multiple factors, complicating its interpretation [80–82]. Factors identified include age, comorbidities, sex, race, marital status and comorbidities [80–82]. Of the 38 studies included, only 20 accounted for confounding factors. This limits conclusions that can be drawn from the current literature. The relationship between biomarkers and symptoms is likely multifactorial which makes it challenging for studies to include all potential confounders. Nonetheless, future studies should attempt to adjust for potential major confounders in analyses or exclude them to enable a better understanding of the relationship between NLR and symptoms. Lastly, most studies in this review (*n* = 31) were retrospective which may introduce selection bias and affect data quality. Future research should include longitudinal studies investigating the relationship between these biomarkers and symptoms or toxicities as a primary outcome to further elucidate the relationship between the outcomes of interest.

## Conclusion

The review highlights the associations between blood biomarkers and symptoms or toxicities and sheds light on the current inconsistencies within the literature. NLR shows the strongest association with symptoms or toxicities, and further prospective studies should be conducted to determine specific NLR cut-off values and the relationship with patient-reported symptoms. However, due to the heterogeneity of studies in this review and inconsistent end points utilised, it is difficult to determine clear recommendations for use of NLR, PLR and MLR with symptoms, toxicities and QoL in clinical practice.

## Data Availability

No datasets were generated or analysed during the current study.
